# A novel mutation in *TTC8* is associated with progressive retinal atrophy in the golden retriever

**DOI:** 10.1186/2052-6687-1-4

**Published:** 2014-04-16

**Authors:** Louise M Downs, Berit Wallin-Håkansson, Tomas Bergström, Cathryn S Mellersh

**Affiliations:** Kennel Club Genetics Centre, Animal Health Trust, Lanwades Park, Newmarket, UK; The Swedish Kennel Club (SKK), Stockholm, Sweden; Department of Animal Breeding and Genetics, Swedish University of Agricultural Sciences (SLU), Uppsala, Sweden

## Abstract

**Background:**

Generalized progressive retinal atrophy (PRA) is a group of inherited eye diseases characterised by progressive retinal degeneration that ultimately leads to blindness in dogs. To date, more than 20 different mutations causing canine-PRA have been described and several breeds including the Golden Retriever are affected by more than one form of PRA. Genetically distinct forms of PRA may have different clinical characteristics such as rate of progression and age of onset. However, in many instances the phenotype of different forms of PRA cannot be distinguished at the basic clinical level achieved during routine ophthalmoscopic examination. Mutations in two distinct genes have been reported to cause PRA in Golden Retrievers (prcd-PRA and GR_PRA1), but for approximately 39% of cases in this breed the causal mutation remains unknown.

**Results:**

A genome-wide association study of 10 PRA cases and 16 controls identified an association on chromosome 8 not previously associated with PRA (p_raw_ = 1.30×10^-6^ and corrected with 100,000 permutations, p_genome_ = 0.148). Using haplotype analysis we defined a 737 kb critical region containing 6 genes. Two of the genes (*TTC8* and *SPATA7*) have been associated with Retinitis Pigmentosa (RP) in humans. Using targeted next generation sequencing a single nucleotide deletion was identified in exon 8 of the *TTC8* gene of affected Golden Retrievers. The frame shift mutation was predicted to cause a premature termination codon. In a larger cohort, this mutation, *TTC8*_c.669delA_, segregates correctly in 22 out of 29 cases tested (75.9%). Of the PRA controls none are homozygous for the mutation, only 3.5% carry the mutation and 96.5% are homozygous wildtype.

**Conclusions:**

Our results show that PRA is genetically heterogeneous in one of the world’s numerically largest breeds, the Golden Retriever, and is caused by multiple, distinct mutations. Here we discuss the mutation that causes a form of PRA, that we have termed PRA2, that accounts for approximately 30% of PRA cases in the breed. The genetic explanation for approximately 9% of cases remains to be identified. PRA2 is a naturally occurring animal model for Retinitis Pigmentosa, and potentially Bardet-Biedl Syndrome.

**Electronic supplementary material:**

The online version of this article (doi:10.1186/2052-6687-1-4) contains supplementary material, which is available to authorized users.

## Lay summary

***Progressive retinal atrophy*** (PRA) is a group of inherited eye disorders that occur in many different dog breeds. Each form of PRA shows a simple pattern of ***mendelian inheritance***, and is due to a mutation in one gene. However, over 20 different ***PRA-causing mutations*** have now been identified in a number of different genes. Some breeds, including Golden Retrievers (GR), may have more than one genetic form of PRA.

Canine PRA is considered to be the equivalent of ***Retinitis Pigmentosa*** (RP), which is a group of inherited human eye diseases.

This paper has identified a gene called ***TTC8*** that is associated with PRA in GR. Interestingly the human TTC8 gene has previously been associated with RP. There is a one DNA base deletion in the gene which results in a shorter than normal protein to be made by the faulty version of the gene.

All the cases in this study were genetically screened for the other two known mutations for PRA in GR, and none were homozygous (two copies) for either of those mutations.

The gene was first identified by whole genome scanning 10 cases and 16 controls. A single mutation was identified in the gene by DNA sequencing, and it was confirmed by screening a larger number of dogs.

## Background

In animals inherited and progressive retinal diseases are commonly referred to as progressive retinal atrophy (PRA), which is characterised by a progressive bilateral retinal degeneration resulting in loss of vision. In typical PRA rod photoreceptor responses are lost first followed by cone photoreceptor responses [1]. Fundus changes observed in PRA are bilateral and symmetrical and include tapetal hyper-reflectivity in the early stages followed by vascular attenuation, pigmetary changes and atrophy of the optic nerve head in the later stages of disease [2]. Numerous, genetically distinct, forms of PRA have been documented in more than 100 dog breeds and while they exhibit similar clinical signs, the aetiology, age of onset and rate of progression may vary both between and within breeds. While a particular mutation (and corresponding form of PRA) may be shared by multiple breeds, due to the population structure of the domestic dog most PRA-affected dogs within a single breed are expected to share the same mutation. At least 20 disease-causing mutations have so far been associated with PRA. Most of them are autosomal recessive diseases but there are examples of X-linked as well as dominant PRA-disorders in dogs (for review see [3]). However, the causative mutation for many forms of PRA remains undefined. PRA is considered the veterinary equivalent of Retinitis Pigmentosa (RP), which is the collective name for a group of inherited human retinal disorders that leads to progressive loss of vision in approximately 1 in 4000 people [4–6]. Rod photoreceptor cells are predominantly affected and therefore clinical symptoms typically include night blindness and loss of peripheral vision. With disease progression the cones also degenerate resulting in central vision loss and eventually complete blindness is possible. To date, more than 192 genes have been shown to cause a wide spectrum of human retinal disease, including RP [7]. Mutations in these genes currently only account for approximately 30% of autosomal recessive RP cases [8]. RP is also a major component of a number of systemic diseases including Bardet-Biedl Syndrome (BBS).

Canine diseases have already proved valuable natural models for the study of many varied human conditions such as cardiac conotruncal malformations [9], myotubular myopathy [10] and hereditary retinopathies such as Leber congenital amaurosis (LCA) and achromatopsia [11, 12]. Further to this, canine models for human eye diseases have proved invaluable in gene-therapy studies, most notably the canine model of LCA associated with *RPE65*[13–17].

Most PRA cases in the Golden Retriever (GR) are clinically indistinguishable from PRA cases of other breeds. The mode of inheritance appears from pedigree information to be consistent with autosomal recessive and the age of diagnosis is most commonly at approximately 5 years of age [18]. We previously identified a form of PRA, GR_PRA1, caused by a mutation in the *SLC4A3* gene that accounts for the majority (61%) of cases of PRA in the GR breed [18]. In the closely related Labrador Retriever (LR) breed, a mutation in the *PRCD* (progressive rod cone degeneration) gene can explain the majority of PRA cases [19]. The PRCD-mutation has also been associated with PRA in a small number of PRA-affected GRs [18].

Here we report the identification of a single base deletion in the *TTC8* gene*,* which is one of seven genes encoding a protein complex (BBSome) that has been proposed to promote ciliary membrane biogenesis and to be an important factor in the development of Bardet-Biedl Syndrome [20]. The deletion causes a shift in the reading frame resulting in a subsequent premature termination codon. We present evidence that this putative loss-of-function mutation represents a third susceptibility locus for PRA, known hereafter as GR_PRA2, in Golden Retrievers.

## Results

### PRCD and PRA1 screening

To exclude the possibility that the affected GRs were positive for the mutations already known to cause prcd-PRA or GR_PRA1, all 29 GR cases in our study were screened for the previously described, autosomal recessive PRCD [19] and GR_PRA1 mutations [18]. None of the affected GRs were homozygous for either of these mutations. A single individual was heterozygous for the PRCD mutation and five were heterozygous for the GR_PRA1 mutation, while the remaining 23 were homozygous for the wildtype (normal) alleles at both loci.

### Genome-wide association mapping

**Figure 1:**
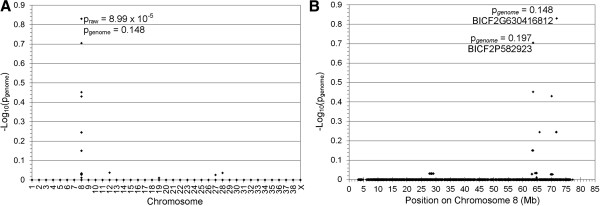
**Genome-wide association mapping of PRA in Golden Retrievers.** -Log_10_ of p-values after correction for multiple testing and population stratification with 100,000 permutations and IBS clustering, respectively. **(A)** -Log_10_ plot of genome-wide association results show a single, albeit not statistically significant, signal on CFA8 (p_raw_ = 8.99 × 10^-5^, p_genome_ = 0.148). The most significant of the raw and permuted values are indicated. **(B)** The associated SNPs on CFA8 form two distinct signals with the most associated SNPs at 63.614 Mb and 71.732 Mb. Permuted values at these loci are indicated.

Ten PRA-affected Golden Retrievers and 16 healthy controls (all but two of which were over the age of seven years when last examined) were genotyped with the 170 k CanineHD BeadChips (Illumina). After filtering, 103,264 SNPs were used in a Genome-wide association (GWA) analysis and a genome-wide significant association was found on chromosome 8 (CFA8; p_raw_ = 1.303×10^-6^, p_genome_ = 0.036). Identity-by-state (IBS) clustering using genome-wide SNP marker data confirmed the presence of population stratification with a genomic inflation factor > 1 (λ = 1.32). After correction for stratification by analysing for association with a CMH meta-analysis the signal on CFA8 remained the strongest signal (p_raw_ = 8.99×10^-5^) and the inflation factor was reduced (λ = 0.86) (Figure 1). While the signal dropped below the level of significance after correcting for multiple testing (p_genome_ = 0.148; Figure 1), the reduction of the inflation factor to a value <1 suggests an over-correction. The signal on CFA8, nevertheless remained the only associated region observed anywhere on the genome and extended from 63.600 to 71.732 Mb with the most significantly associated SNP, BICF2G630416812 (p_genome_ = 0.148) at 71.732 Mb. Further investigation of the signal on CFA8 revealed that it was formed by two apparently independent and distinct signals with the most highly associated SNPs at 63.614 Mb (BICF2P582923; p_raw_ = 1.16×10^-4^; p_genome_ = 0.197) and 71.732 Mb (BICF2G630416812; p_raw_ = 8.99×10^-5^; p_genome_ = 0.148) respectively (Figure 1). These SNPs are hereafter referred to as Marker 1 and Marker 2 respectively. A second analysis using Fast Mixed Model to correct for population stratification generated results that were consistent with those described above, conveying support for the CMH meta-analysis (data not shown).

### Haplotype and homozygosity analysis

**Figure 2:**
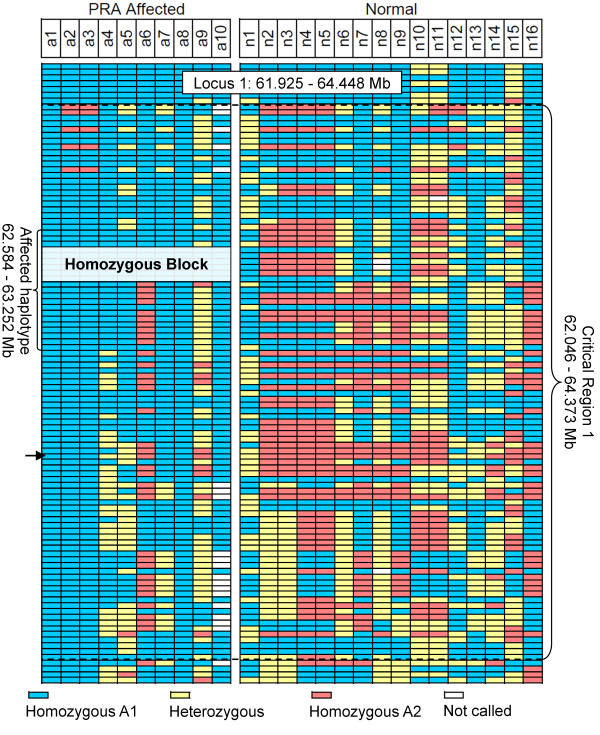
**Critical region definition using homozygosity analysis.** SNP genotypes for 10 PRA cases and 16 PRA controls, over one of the two regions identified during the GWA study. The most associated SNP in the region, BICF2P582923 (Marker 1) at 63.614 is indicated with an arrow. All 10 cases, as well as 5 of the controls, share a 437 kb homozygous block, while 8 cases and none of the controls share a larger homozygous region (Affected haplotype) upstream of Marker 1.

**Figure 3:**
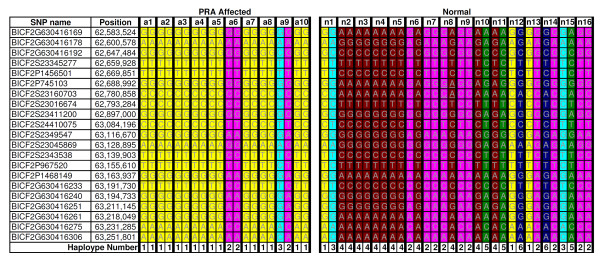
**Fine mapping using haplotype analysis.** SNP genotypes for 10 PRA cases and 16 PRA controls, over the 668 kb “Affected haplotype” identified during the GWA study. Inferred phasing of the 21 SNP markers revealed six unique haplotypes. Haplotype number 1 (yellow) is homozygous in 8/10 cases, but none of the controls.

SNP homozygosity analysis of the region between Marker 1 or Marker 2 did not reveal a haplotype that was homozygous in all cases and no controls (data not shown). In a region 500 kb upstream of Marker 1 on CFA8 (62.647 to 63.084 Mb) a 437 kb region was identified for which all of the PRA cases were homozygous (Homozygous Block, Figure 2). However, five of the controls were also homozygous for the “affected” haplotype in this region. There was also a larger region comprising 21 SNPs over which the haplotypes of most of the cases were different from those of the controls (Affected haplotype, Figure 2). Inferred phasing of these SNP markers, spanning 668 kb, revealed the presence of six haplotypes, one of which was homozygous in 8/10 cases but none of the controls (Haplotype number 1 in Figure 3). As this putative PRA-associated haplotype does not include Marker 1 or Marker 2, broad critical regions were defined to ensure sequence coverage of Markers 1 and 2 and the PRA-associated haplotype. The critical regions therefore extend from 62.046 to 64.373 Mb (Critical region 1) and 71.147 to 72.634 Mb (Critical region 2), defining regions of 2.327 and 1.487 Mb respectively, and 3.814 Mb in total. Critical region 1 contains 17 genes, 14 of which have human orthologues. Two genes in the region have previously been associated with inherited retinopathies in humans; Spermatogenesis associated protein 7 (*SPATA7*) has been associated with juvenile RP and LCA [21], and tetratricopeptide repeat domain 8 (*TTC8*) with RP as well as Bardet-Biedl Syndrome (BBS) [22–24]. Critical region 2 contains 11 genes, all of which have human orthologues, none of which have previously been associated with retinal disease in any species.

### Sequencing

**Figure 4:**
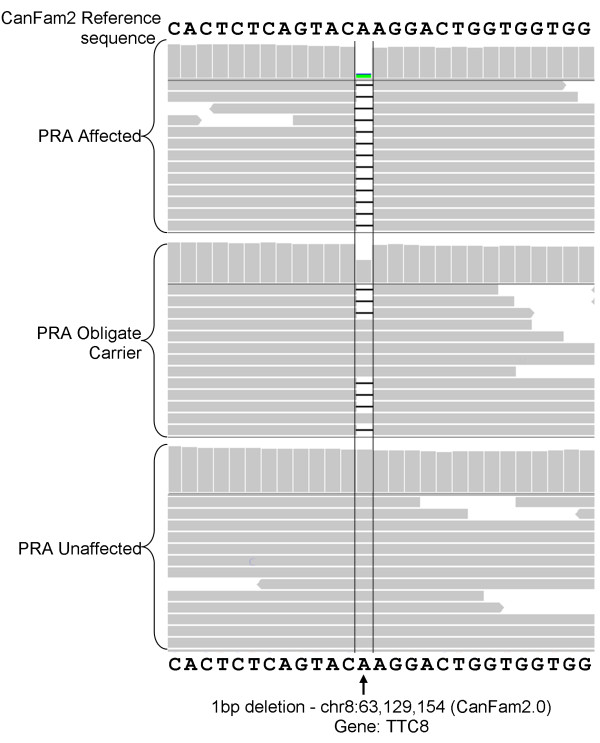
**IGV display of the 1-bp deletion in TTC8 (c.669delA).** Each of the three samples (PRA-affected, obligate carrier and control) viewed in IGV are represented by two panels. The upper panel is a histogram where the height of each column is representative of the read depth at that location. The lower panel is a graphical view of some of the reads that align to that location. The “A” base indicated is absent in almost all reads in the PRA-affected sample, approximately half the reads in the obligate carrier and sample and none of the reads in the PRA-unaffected (control) sample.

To identify potential disease-causing mutations we undertook targeted resequencing of both critical regions in 10 dogs (five affected, two obligate carrier and three control dogs). Repetitive DNA elements, making up 41% of the regions, were masked during the design of custom RNA baits and as a result approximately 59% of the 3.814 Mb critical region was enriched and sequenced. More than 287 million reads were generated across all 10 DNA samples (representing a 14.7 Gb dataset), of which 73% were mapped to the targeted regions on CFA8. Approximately 74% of the regions covered by RNA baits were sequenced with more than 30× read coverage, and the average read depth across the targeted region for each sample ranged from 230× to 329×. We identified more than 25,000 SNPs and 2,400 indels when compared with the CanFam2 reference sequence. Of these 666 SNPs and 168 indels segregated with the phenotype. Two provocative variants were identified that were predicted to alter the protein product. One was a non-synonomous substitution in exon 12 of the *SPATA7* gene (CFA8: 62,735,867 bp; c.A1378G), resulting in a missense mutation (p.Thr460Ala). The other provocative variant was a single base (adenine) deletion in exon 8 of *TTC8* (CFA8: 63,129,154; c.669delA; Figure 4).

### Transcript evaluation

All of the coding sequence of the *SPATA7* and *TTC8* retinal transcripts from a healthy dog of unknown age or breed were successfully sequenced, confirming that both genes are transcribed in the canine retina. The presence of the *SPATA7* variant, c.A1378G, in the healthy retinal mRNA transcript was surprising. While there is a chance the tissue could have come from a dog that had not yet developed PRA, we think it unlikely. The location of this variant was only modestly conserved at the DNA level and poorly conserved at the amino acid level in 35 eutherian mammals (data not shown). In addition the c.A1378G variant was predicted to be benign by SIFT [25] and PolyPhen [26]. Taken together these data suggested that the variant was unlikely to be pathogenic and it was therefore eliminated from further investigation.

**Figure 5:**
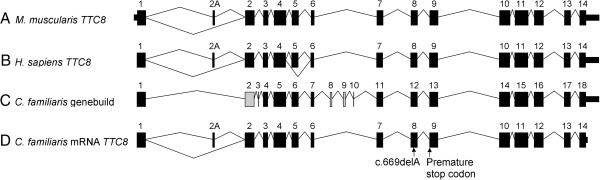
**Graphical comparison of the exons and intron-exon boundaries of human and canine TTC8. (A)** Mouse (Mus muscularis) *TTC8*. **(B)** Human (*Homo sapien*) *TTC8*. **(C)** Canine (*Canis familiaris*) *TTC8* as predicted by Ensembl genebuild. Thirteen of the genebuild exons predicted are identical to the human exons (black). Exon 2 (grey) is different at the 3′ intron-exon boundary. Exons 3, 8, 9 and 10 (grey) show no sequence or size similarity to their human equivalents and are probably incorrect. **(D)** Canine *TTC8* exons confirmed by sequencing the retinal mRNA transcript. Exon 2A has not been predicted by Ensembl genebuild. The location of the sequence variant is indicated.

**Figure 6:**
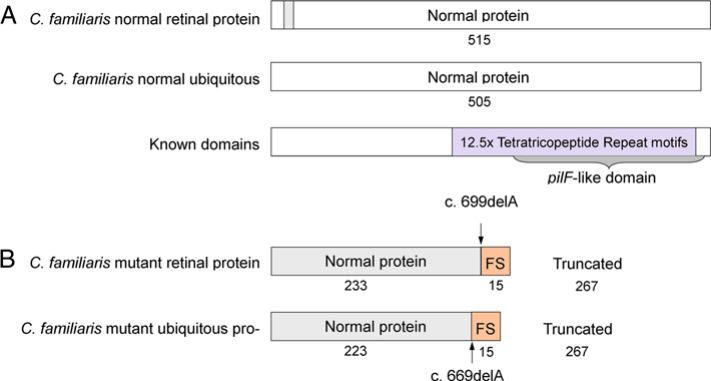
**Effect of** ***TTC8***_**c.669delA**_ **on the protein. A)** The normal retina-specific and ubiquitous isoforms encode 515 and 505 amino acids respectively. The proteins differ only with the presence or absence of 10 amino acids unique to the retina-specific isoform (grey). The remainder of the protein is identical in both isoforms. **B)** Both isoforms are affected by the c.669delA variant. 233 amino acids at the N-terminus of the retina-specific protein and 223 of the ubiquitous isoform are normal. However the deletion causes a shift in the reading frame of 15 amino acids, leading to a premature termination codon. This results in a truncated protein product, lacking 267 residues of the C-terminus of both isoforms. More than half of the protein is therefore absent.

Our sequencing data indicated that two isoforms of *TTC8* are transcribed in the canine retina and intron-exon boundaries are identical to those of the human and mouse (Figure 5), which is in conflict with the boundaries predicted by Ensembl genebuild for the canine gene. We were unable to sequence the full 5′ and 3′ UTRs for any isoform. From the sequencing of the retinal mRNA transcripts of both isoforms we discovered that canine *TTC8* (Genbank accession no JQ941743) contains 505 amino acids and *TTC8*_2A_ (Genbank accession no JQ941742) contains 515 amino acids (Figure 6), with molecular weights of 57.571 kDa and 58.247 kDa respectively, predicted using the ExPASy Proteomics Server [27].

The exonic variant detected by resequencing was a frame-shifting deletion of a single adenine in exon 8 (*TTC8*_c.669delA_ in transcript ENSCAFT00000050179, CFA8:63,129,154). The deletion was predicted to cause a premature stop codon (p.Lys223ArgfsX15), possibly resulting in the degredation of mRNA by nonsense-mediated decay or a truncated protein product (Figure 6). *TTC8*_c.669delA_ affects both isoforms of the protein.

### Mutation screening

We screened all the 26 GR dogs (10 cases and 16 controls) that were included in the GWA study for the coding variant, *TTC8*_c.669delA_, to confirm the association of this variant with PRA and compare it with the most highly associated SNP markers, Marker 1 and Marker 2. The variant showed significant allelic association with PRA (p_raw_ = 6.31 × 10^-7^, p_genome_ = 0.019) and was more strongly associated than both Marker 1 at 63.614 Mb (p_raw_ = 5.79 × 10^-6^, p_genome_ = 0.109) and Marker 2 at 71.732 Mb (p_raw_ = 1.30×10^-6^, pP_genome_ = 0.037). Eight out of ten PRA cases and none of the controls were homozygous for *TTC8*_669delA_. Both of the two remaining cases were homozygous for the wildtype (normal) haplotype. Neither were either of these two cases homozygous for the minor allele at Marker 2; one was heterozygous and the other homozygous for the wildtype/major allele. While the variant showed incomplete association with PRA, it has a strong likelihood of a deleterious effect on the protein. In addition, the nucleotide and the amino acid affected by *TTC8*_c.669delA_ is conserved in 32 eutherian mammals (data not shown). This mutation is therefore highly likely to be the causal mutation for GR_PRA2. Analysis of the segregation of *TTC8*_c.669delA_ with PRA in a family of Swedish ancestry (Additional file 1: Figure S1) indicated that PRA2 is recessive and fully penetrant.

We screened 2474 additional GR dogs, making a total of 2500 GRs (29 PRA affected that were not caused by GR_PRA1 or PRCD, five obligate carriers, 459 unaffected dogs of any age and 2007 dogs of unknown clinical status) tested for the *TTC8*_c.669delA_ mutation (Table 1), to confirm that it is not a commonly occurring polymorphism in this breed. Of the 29 PRA cases included in the study 22 (75.9%) were homozygous for *TTC8*_c.669delA_ (*TTC8*^-/-^) and all 464 dogs known to be clinically free of PRA at their last eye examination, including five obligate carriers of PRA, were either carriers of the mutant allele (3.9%; *TTC8*^+/-^) or homozygous for the wildtype allele (96.1%; *TTC8*^+/+^). The mutation described here accounts for PRA cases from the UK, Sweden, France, Finland and USA. A subset of the complete sample cohort were used to calculate allele frequencies in populations within five countries (Table 2).

**Table 1:** **PRA2 genotypes and PRA clinical status for 2500 GRs**

	PRA clinical status				
Genotype^i^	PRA case	PRA obligate carrier	Clear	Unknown	Total
**TTC8** ^**-/-**^	22 (75.9%)	0 (0%)	0 (0%)	4 (0.2%)	26
**TTC8** ^**+/-**^	1 (3.4%)	2 (40.0%)	16 (3.5%)	130 (6.5%)	149
**TTC8** ^**+/+**^	6 (20.7%)	3 (60.0%)	443 (96.5%)	1873 (93.3%)	2325
**Total**	29	5	459	2007	2500

^i^The wildtype allele is represented by “+” and the mutant allele by “-”.

**Table 2:** **Frequency of** ***TTC8***_**c.669delA**_ **in various countries**

	Country of ancestry				
	UK	Sweden	Denmark	France	USA
**Number tested**	88	736	132	87	179
***TTC8***_**c.669delA**_ **frequency**	0.011	0.042	0.045	0.034	0.011
**1 in # affected expected**	7700	560	480	840	8000
**1 in # carriers expected**	45	12	12	15	45

To determine whether *TTC8*_c.669delA_ is associated with PRA in related breeds we screened a further 175 dogs from three closely related breeds that could realistically share polymorphisms with the GR; 48 Chesapeake Bay Retrievers (CBR), 59 Flat-Coat Retrievers (FCR) and 71 LRs, including 19 LRs and 2 CBRs that were clinically affected with PRA but that were clear of the prcd mutation. One LR with PRA was homozygous for the mutation (*TTC8*^-/-^). The remaining 70 LR, 45 CBR and 59 FCR dogs were homozygous wildtype (*TTC8*^+/+^).

### Additional phenotyping

One of the GRs with PRA in our cohort that was homozygous for *TTC8*_c.669delA_ was reported to have clinical signs in addition to PRA. These were described as “hormonal changes and thyroid problems” by the dog’s owner/veterinarian, the precise nature of which are unknown, and attempts to gather further information pertaining to the phenotype of this dog were unsuccessful.

Assessment of systemic clinical signs in additional dogs that were homozygous for *TTC8*_c.669delA_ was attempted, by means of an owner-completed questionnaire, but unfortunately only 2/10 were completed and returned:

1. A bitch, diagnosed with PRA at the age of 5.9 years was reported to be in good health. She was short for the breed, at 30 kg was overweight for her size and gained weight easily. She had undergone metabolism tests (details of which were not reported), and while not treated, the results were considered borderline abnormal. The owner also reported that the dog had never been able to catch a ball in the air, suggesting it had poor coordination.
2. A dog, diagnosed with PRA at 2.9 years, was reported to be in good health until his death at 9 years, from cancer. At 52 cm tall, he was considered small for the breed, but was not overweight. The owner reported two behavioural idiosyncrasies: he was aggressive towards certain people when in the home, but not outside, and he also appeared to have a poor sense of smell.

## Discussion

In Golden Retrievers, mutations in two genes (*SLC4A3* and *PRCD*) causing PRA in the breed have been described [18, 19]. These mutations account for approximately 61% of cases of PRA [18]. The PRCD mutation appears to be very rare in the breed [28]. Indeed, we have not identified any dogs homozygous for the PRCD mutation, and less than 2% are carriers of the mutation. Using a GWA analysis approach, we have identified a third causal mutation for PRA in the GR, a novel mutation in the *TTC8* gene. We found that while this mutation does not explain all remaining cases of PRA in our study, suggesting that there is at least one more genetically distinct form of PRA in this breed, it does appear to be fully penetrant and a common cause of PRA in the breed.

Sequencing of *SPATA7* and *TTC8* from healthy retinal mRNA served four purposes: 1. It confirmed the presence of both mRNA transcripts in the normal canine retina. 2. The presence of the *SPATA7*_c.A1378G_ variant in mRNA from a healthy dog allowed the elimination of this variant from further investigation. 3. It revealed that the intron-exon boundaries predicted by the CanFam2 annotation for *TTC8* in the dog are incorrect for five exons. They are instead identical to the human and mouse boundaries (Figure 5). 4. It revealed an exon orthologous to human exon 2A, that is absent from the Ensembl canine (CanFam2) prediction (Figure 5). As is the case in humans and mice, canine *TTC8* is alternatively spliced to produce two isoforms (*TTC8* and *TTC8*_2A_). The precise functional difference between the two isoforms is unknown, but it is thought TTC8_2A_ plays in important role in the function of the protein in the photoreceptor cell-containing outer nuclear layer of the retina [23].

In order to further test the validity of the insertion mutation, we screened 2500 GRs for the mutation (Table 1). We found that 75.9% of the PRA cases (not caused by PRCD or GR_PRA1), 40% of the obligate PRA carriers and 100% of clinically unaffected dogs (which could be clear of the mutation or carry a single copy) have *TTC8* genotypes that are concordant with their clinical status. All 22 dogs with known phenotypes and homozygous for the mutation i.e. *TTC8*^-/-^, have developed PRA, suggesting that the mutation is fully penetrant within the Golden Retrievers investigated. The inheritance observed in a family of eight dogs (three cases) is supportive of a recessive mode (Additional file 1: Figure S1). The presence of the variant in GR dogs from countries including the USA, UK, France, Denmark and Sweden suggests the variant may have arisen prior to the geographic dispersion of the breed. The mutant allele frequencies indicate that between 1 in 480 and 1 in 8000 GRs is likely to be affected with this form of PRA, although up to 1 in 12 are expected to carry the mutant gene. There is a group of dogs with genotypes discordant with their phenotypes, comprising 7 PRA-affected dogs that are not homozygous for *TTC8*_c.669delA_ and three obligate carriers that do not carry *TTC8*_c.669delA_. It is formally possible that the mutation has a dominant mode of inheritance with incomplete penetrance, or complex trait or compound heterozygote effects, although we have no evidence to suggest this might be the case. Indeed, only 1/7 PRA-affected dogs that are not homozygous for *TTC8*_c.669delA_ is heterozygous and could therefore potentially be a compound heterozygote. A further three of these seven dogs are heterozygous at the GR_PRA1 (*SLC4A3*) locus, and PRA in these dogs could potentially be caused by compound heterozygosity in *SLC4A3*. However, the three remaining dogs are homozygous for the wildtype alleles at all three loci described to date i.e. *SLC4A3*, *TTC8* or *PRCD*, suggesting a fourth locus must be causing PRA in these dogs. The observation that none of these seven dogs are heterozygous at more than one locus suggests that the additive effects of heterozygosity is unlikely to be the cause of PRA in these dogs. Given that three distinct loci have now been implicated in PRA in the breed, these data taken together suggest it is likely that still more loci are responsible for the discordant cases.

The absence of the mutant *TTC8* allele from FCR and CBR dogs tested, including some dogs affected with PRA, indicates that the mutation is rare and probably mainly confined to the GR breed, although identification of a LR (with clinically apparent PRA) homozygous for the variant suggests it may be present in the LR breed as well. However, as only 1/19 LR PRA cases, all of which have previously tested clear for prcd, is caused by the *TTC8* variant, it is clearly a minor cause of PRA in the breed.

The PRA cases in our study that were homozygous for the *TTC8* variant had an average age of diagnosis of 4.51 years, while the discordant GR PRA cases i.e. *TTC8*^+/+^ and *TTC8*^+/-^ had an average age at diagnosis of 6.46 years. This difference could be indicative of the segregation of a fourth form of PRA in the GR breed, with a slightly older age of onset than GR_PRA2, although the age at diagnosis may not necessarily accurately reflect the age of onset.

**Figure 7:**
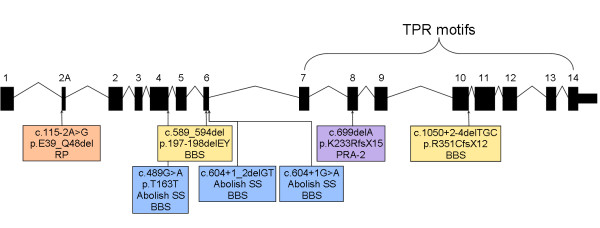
**Comparison of human and canine retinal disease mutations.** In humans, only mutations in exon 2A cause RP (orange [23]), while mutations elsewhere have been associated with BBS (yellow [22] and blue [24]). *TTC8*_c.669delA_ (purple) is associated with PRA in GRs.

*TTC8* (a.k.a. *BBS8*) encodes the protein tetratricopeptide repeat domain 8 and was recognised as a candidate gene due to its previous implication in BBS and autosomal recessive RP in humans [22–24]. TTC8 is one of seven BBS proteins that form a stable complex known as the BBSome, which functions primarily at the ciliary membrane and is thought to play a role in ciliogenesis [20]. BBS is a pleiotropic disease and typical symptoms include obesity, retinal degeneration, kidney malformation, olfactory deficits, polydactyly, learning disabilities and ataxia or poor coordination. While the syndrome is usually inherited in an autosomal recessive manner, triallelic inheritance has also been observed [29]. Human *TTC8* is made up of 14 exons and alternative splicing of the fifth exon results in two isoforms that are widely expressed [22]. In addition a retina-specific isoform, localized to the outer nuclear layer, is created by the alternative splicing of exon 2A [23]. While PRA is widely considered to be the veterinary equivalent of RP, the limited characterisation of PRA at a cellular level is insufficient to fully justify this comparison. Further investigations are required to understand the cellular processes involved in this form of PRA, including whether the rod or cone photoreceptor cells are affected first. While most mutations that significantly alter the structure of TTC8 cause BBS in humans, those that result in in-frame deletion or skipping of the 10 amino acids encoded by exon 2A cause non-syndromic RP (Figure 7). The *TTC8*_c.669delA_ variant in the GR is predicted to have a significant effect on the structure of the protein, including the loss of all tetratricopeptide repeat (TPR) motifs near the carboxyl terminus (Figure 6), known to be protein-protein interaction motifs [22]. Most of the affected dogs, however, presented with typical PRA and no additional clinical signs that we were made aware of. It is possible that other clinical signs in the dog have simply not been reported to, or by, the examining ophthalmologists. One dog with PRA2 was reported to have “hormonal changes and thyroid problems”, the precise nature of which are unknown. Attempts to gain further information pertaining to this dog have thus far been unsuccessful. Signs seen in two other dogs (small stature, obesity, aberrant metabolism and olfaction, and unusual behaviour), as reported by their owners, suggest there may be more to the phenotype of PRA2 than simply PRA. As the signs reported are subjective, an objective, clinical investigation is warranted. Attempts to conduct a clinical examination of these dogs have been unsuccessful as the dogs were unavailable. Nevertheless, it is clear there are phenotypic differences between human BBS and canine PRA2, as the variety of symptoms that are seen in human patients are not seen in the dog. It is unclear whether these phenotypic differences are purely due to species-specific differences or some other as yet unknown mechanism, such as the effect of modifier genes. The TTC8 protein is clearly critical to ensure efficient function of ciliated tissue in humans, as demonstrated by the BBS phenotype. In contrast, it may be that the TTC8 protein is critical for photoreceptor function in dogs, but less so in other tissues, perhaps due to compensatory effects of other proteins. This would explain a retina-specific or possibly less severe systemic phenotype in dogs. Alternatively, in non-retinal tissues there may be other proteins that compensate for the loss of TTC8 in dogs more than in humans.

## Conclusions

The identification of a frameshift deletion in *TTC8* in GR dogs with PRA, that is likely to be a significant susceptibility locus for PRA in this breed, establishes PRA2 as a model for human RP, and potentially BBS. This form of PRA in the GR may prove to be a valuable model for further studies to enhance our understanding of visual pathways and gene therapy investigations.

## Methods

### Sample processing

The diagnosis of individual dogs was provided by veterinary ophthalmologists through the BVA/KC/ISDS (British Veterinary Association/Kennel Club/International Sheep Dog Society) Eye Scheme in the UK, the Swedish Kennel Club Eye Scheme in Sweden and independent veterinary ophthalmologists. Cases were defined as dogs diagnosed as affected with PRA i.e. displaying ophthalmoscopic signs of PRA including tapetal hyperreflectivity and vascular attenuation and controls as those free of inherited eye disease of any kind, and at least 7 years old at the time of examination for the GWA analysis or any age for subsequent investigations.

Blood samples were collected into EDTA tubes and genomic DNA was either extracted manually from peripheral blood leukocytes using QIAamp DNA Blood Midi Kit (Qiagen, Hilden, Germany) or automatically on a QIAsymphony SP/AS instrument (Qiagen, Hilden, Germany). DNA was also extracted from whole blood using a Nucleon Genomic DNA Extraction Kit (Tepnel Life Sciences, Manchester, UK), according to the manufacturer’s instructions. For samples collected as buccal mouth swabs, DNA was extracted using a QIAamp® DNA Blood Midi Kit (Qiagen, West Sussex, UK). A canine retinal tissue sample from a dog of unknown breed and free of PRA was taken post mortem, with the owner’s consent. RNA was extracted using an RNeasy Protect Mini Kit (Qiagen, West Sussex, UK) according to the manufacturer’s instructions.

### PRCD and PRA1 screening

We genotyped DNA from 29 PRA-affected GRs for the PRCD and PRA1 mutations. The former was performed using the TaqMan allelic discrimination technique (Applied Biosystems Inc., Foster City, CA) according to the manufacturer’s instructions. Primers (Forward: 5′-GGCCTTTCTCCTGCAGACT-3′; Reverse: 5′-CAGCTTCTCACGGTTGGAC-3′) and PrimeTime Dual-Labelled Probes (G-probe: 5′-FAM-AGCCATGTGCACCACCCTCT-BHQ-3′ and C-probe: 5′-HEX-TGAGCCATGTACACCACCCTCT-BHQ-3′; IDT, Glasgow, UK) were designed with Primer3 [30]. PCR amplification and allelic discrimination plate read and analysis were carried out on a Techne Quantica Real Time Thermal Cycler with the Quansoft software (Bibby Scientific Limited, Staffordshire, UK). The PRA1 genotyping was performed by PCR amplification using fluorescent primers (Forward: 5′-6-FAM-AGAGCAACCTTGTAACCCGTA-3′ and Reverse: 5′-GGAAGAAGGCAATGAGAAAGG-3′; IDT, Glasgow, UK) and subsequent fragment length polymorphism detection using an ABI 3130xl DNA Analyzer and GeneMapper® Software (Applied Biosystems, Inc., [ABI], Foster City, CA).

### Genome-wide association mapping

CanineHD BeadChips (Illumina) were used to obtain genotype calls for 173,662 SNPs using DNA from 10 GR PRA cases and 16 GR controls and GWA analysis was conducted using the software package PLINK [31]. After removing SNPs with a minor allele frequency <5% and missing genotype calls >10% from the analysis, a final data set of 103,264 markers remained. Sample call rate was >99.7% for all samples. Identity-by-state (IBS) clustering and Cochran–Mantel–Haenszel (CMH) meta-analysis with PLINK were used to examine and adjust for population stratification. As a correction for multiple testing, we repeated the GWA analyses using the Max(T) permutation procedure in PLINK (100,000 permutations, denoted by p_genome_). An additional analysis using Fast Mixed Model (FMM) to correct for population stratification was also undertaken [32]. Haplotype phases were inferred using PHASE [33]. Visual inspection of SNP genotypes and haplotypes across the region was performed to define a homozygous critical region.

### Next generation sequencing

Genomic DNA (3 μg) from 10 GR dogs (five PRA-affected, two obligate carrier and three PRA-clear) was used to prepare DNA libraries for sequencing, using the SureSelectXT Custom MP4 Kit (Agilent Technologies). Each kit contained a custom capture library of 34,097 biotinylated RNA baits, 120 bp in length and designed based on the CanFam2 reference sequence using Agilent Technologies’ eArray tool [34]. Baits were designed to give 2× coverage and exclude repeat-masked regions, resulting in coverage of 59.1% (2.25/3.81 Mb) of the targeted regions. Target enrichment was performed according to the manufacturer’s instructions. Initial shearing of genomic DNA using a Covaris S220 and quality assessment of the final library using a 2100 Bioanalyser was undertaken by The Eastern Sequence and Informatics Hub (EASIH, University of Cambridge). The quantity of the captured library was assessed by quantitative PCR using the KAPA Library Quantification Kit for the Illumina Genome Analyzer Platform (KAPA Biosystems), according to the manufacturer’s instructions.

Paired-end sequencing resulting in 51-bp reads was conducted in a single lane on an Illumina Hiseq 2000, by the High Throughput Group (HTG) at the Welcome Trust Centre for Human Genetics (University of Oxford, UK). Sequence reads were aligned with the canine reference sequence (CanFam 2) using BWA [35], variant (SNP and indel) calls were made using GATK [36] and the aligned reads were visualised using the Integrative Genomics Viewer (IGV) [37]. Variants considered candidates for further investigation were those that occurred in splice sites or resulted in non-synonymous changes to a protein, and that were homozygous in PRA cases, heterozygous in obligate carriers and heterozygous or homozygous for the wildtype allele in controls.

### Sanger sequencing

The exon-intron boundaries of canine *TTC8* and *SPATA7* were defined by producing ClustalW [38] alignments using the Ensembl predicted canine transcripts (*TTC8*: ENSCAFG00000017478; *SPATA7*: ENSCAFG00000017354) and available known mouse (*TTC8*: ENSMUSG00000021013; *SPATA7*: ENSMUSG00000021007) and human (*TTC8*: ENSG00000165533; *SPATA7*: ENSG00000042317) Ensembl transcripts. Primer3 [30] was used to design primers in the exons for the amplification of cDNA as well as to design primers in introns seven and eight for the amplification and sequencing of *TTC8* exon 8 in genomic DNA (Additional file 2: Table S1). *SPATA7* and *TTC8* mRNA sequence was amplified by reverse-transcriptase PCR using SuperScript®II Reverse Transcriptase (Invitrogen) according to the manufacturer’s instructions. To further investigate the remaining variant (*TTC8*_c.669delA_) in a larger dataset exon 8 of *TTC8* was amplified by polymerase chain reaction (PCR) using HotStarTaq Plus DNA Polymerase (Qiagen) in genomic DNA from the 26 GRs that were included in the GWA study. PCR products were purified using Multiscreen HTS-PCR filter plates (Millipore). Amplification products were sequenced on an ABI 3130xl DNA Analyzer using BigDye Terminator v3.1 (Applied Biosystems) and sequence traces were assembled, analyzed and compared using the Staden Package [39]. The variant was analysed for association with PRA and compared with the most associated SNP markers, BICF2P582923 and BICF2G630416812, using the software package PLINK [31].

### Mutation screening

The suggestive causative mutation for PRA2 in exon 8, *TTC8*_*c*.669delA_, was screened in 2500 GRs by PCR amplification using fluorescent primers (Forward: 5′-6-FAM- TGCCCTTTCCACAGAGCAC-3′ and Reverse: 5′- CCATGTCTAAGCCCTTCACAA-3′; IDT, Glasgow, UK) and subsequent fragment length polymorphism detection using an ABI 3130xl DNA Analyzer and GeneMapper® Software (Applied Biosystems, Inc., [ABI], Foster City, CA). The panel of 2500 GRs of any age (including the 26 DNA samples already sequenced), was made up of 29 PRA cases, 5 obligate carriers, 459 clear dogs and 2007 dogs with unknown PRA clinical status. Included in this cohort of 2500 dogs were 88 dogs of breeding age (between one and eight years of age), unrelated at the parent level (from 88 different dams and 88 different sires) and of UK ancestry. Also included were 87 dogs of French ancestry, 736 dogs of Swedish ancestry, 132 dogs of Danish ancestry and 179 dogs of US ancestry, collected specifically for allele frequency estimations. In addition, samples from 175 dogs representing three breeds that are closely related to the GR breed were also included in the mutation screening: LR (n = 71), CBR (n = 45) and FCR (n = 59).

### Additional PRA2 phenotyping

Two dogs with PRA that were homozygous for *TTC8*_c.669delA_ were selected for additional phenotyping. The owners of these dogs were asked to complete a questionnaire assessing the overall health of their dog as well as the presence of systemic clinical signs known to be associated with BBS. These included kidney, reproductive, olfactory and behavioural/social abnormalities, obesity and diabetes mellitus.

## Electronic supplementary material

Additional file 1: Figure S1: Segregation of TTC8_c.669delA_ in a GR family. The segregation of *TTC8*_c.669delA_ and PRA in a GR family of Swedish origin is consistent with an autosomal recessive mode of inheritance. “Age at last test” refers to the age of the dog at its last ophthalmoscopic examination. (PNG 426 KB)

Additional file 2: Table S1: Primers for Sanger sequencing. The sequence of primer pairs used for sequencing of mRNA and DNA, along with the expected amplicon sizes. All primers were used with an annealing temperature of 57°C. (XLS 24 KB)

## Abbreviations

GR: Golden retriever
LR: Labrador retriever
CBR: Chesapeake bay retrievers
FCR: Flat-coat retrievers.
