# Supplementary figures and images for: A novel mutation in TTC8 is associated with progressive retinal atrophy in the golden retriever
Source: Canine Genet Epidemiol. 2014 Apr 16;1:4. doi: 10.1186/2052-6687-1-4 (PMC4574394; doi:10.1186/2052-6687-1-4)

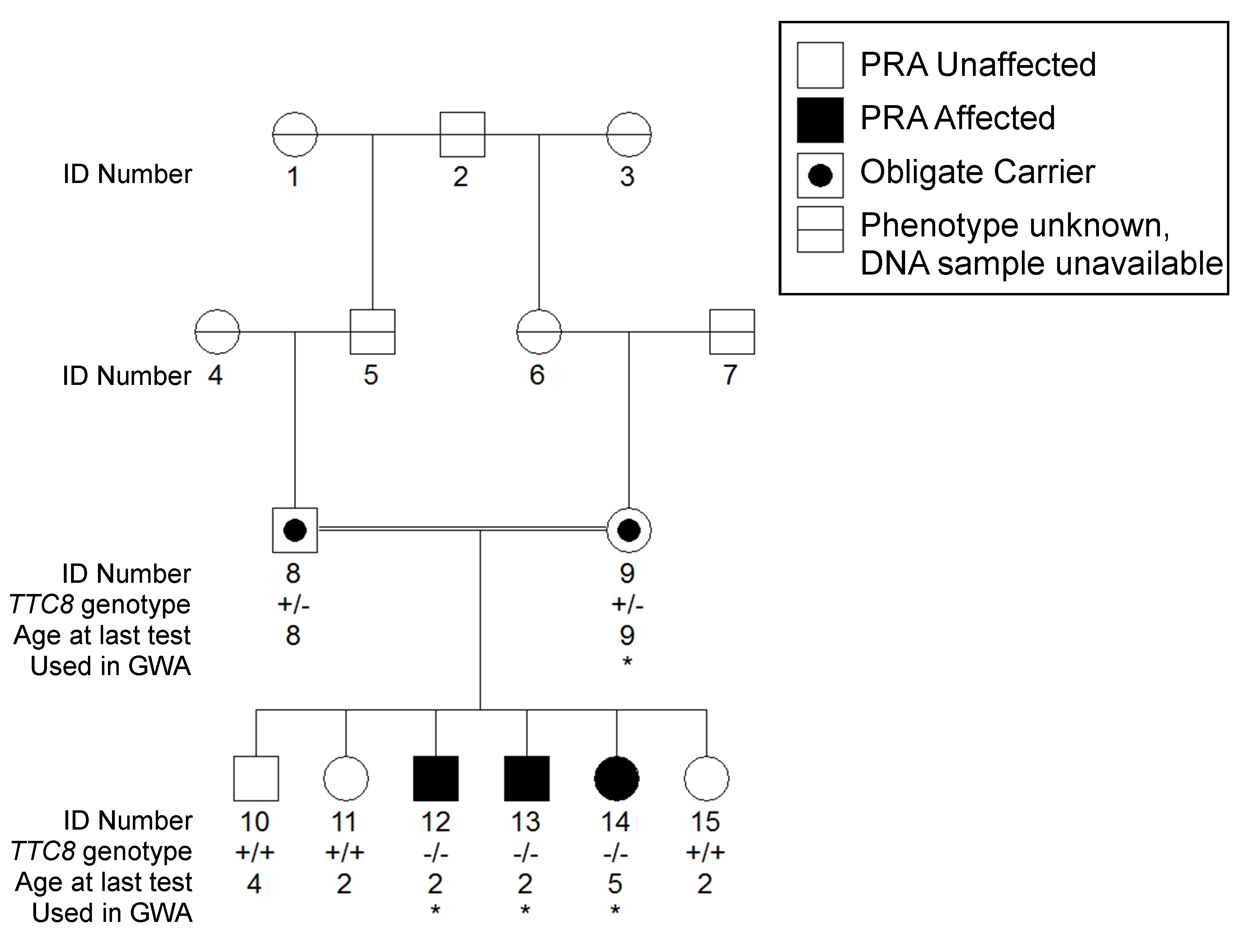

Supplement: Supplementary file 1 — Additional file 1: Figure S1: Segregation of TTC8c.669delA in a GR family. The segregation of TTC8 c.669delA and PRA in a GR family of Swedish origin is consistent with an autosomal recessive mode of inheritance. “Age at last test” refers to the age of the dog at its last ophthalmoscopic examination. (PNG 426 KB) [file 40575_2013_4_MOESM1_ESM.png]
